# Acoustic correlates of body size and individual identity in banded penguins

**DOI:** 10.1371/journal.pone.0170001

**Published:** 2017-02-15

**Authors:** Livio Favaro, Marco Gamba, Claudia Gili, Daniela Pessani

**Affiliations:** 1 Department of Life Sciences and Systems Biology, University of Turin, Turin, Italy; 2 Acquario di Genova, Costa Edutainment SpA, Genoa, Italy; University of Pavia, ITALY

## Abstract

Animal vocalisations play a role in individual recognition and mate choice. In nesting penguins, acoustic variation in vocalisations originates from distinctiveness in the morphology of the vocal apparatus. Using the source-filter theory approach, we investigated vocal individuality cues and correlates of body size and mass in the ecstatic display songs the Humboldt and Magellanic penguins. We demonstrate that both fundamental frequency (*f*_0_) and formants (*F*_1_-*F*_4_) are essential vocal features to discriminate among individuals. However, we show that only duration and *f*_0_ are honest indicators of the body size and mass, respectively. We did not find any effect of body dimension on formants, formant dispersion nor estimated vocal tract length of the emitters. Overall, our findings provide the first evidence that the resonant frequencies of the vocal tract do not correlate with body size in penguins. Our results add important information to a growing body of literature on the role of the different vocal parameters in conveying biologically meaningful information in bird vocalisations.

## Introduction

Animal vocalisations have the potential to provide conspecifics with a variety of information about age, sex, social status [1,2], and even emotional states [3]. Moreover, in several bird and mammals, individuals can be discriminated or recognised by conspecifics using vocalisations [4,5]. The potential to inform about individuality is of particular importance in social species, in which individual recognition is considered to be essential for almost all aspects of social life [6]. However, the mechanisms used by animals to encode individual identity information in calls vary widely. Vocal learners modify the acoustic structure of their vocalisations to generate individually distinctive and unique call types for each group member [7,8]. By contrast, in non-vocal learners, the acoustic features of calls are known to be more fixed and individual variation in vocalisations can originate from distinctiveness in the morphology or size of the vocal apparatus [9,10]. In animals where the acoustic features of vocalisations are linked to anatomical constraints that cannot be faked [11,12], the vocal signal can also provide “honest” information about the emitter [13,14]. Finally, call production is energetically demanding [15,16] and “honesty” in vocal displays can be guaranteed because they are costlier to produce for low-quality individuals (i.e. the “handicap principle” [17]).

The source-filter theory of vocal production [18,19] is a robust framework for studying mammal vocal communication. According to this theory, calls are generated by vibrations of the vocal folds in the larynx (source, determining the fundamental frequency, “*f*_0_”), and are filtered by the supralaryngeal vocal tract (filter, resulting in peaks called “formants”). By contrast, in birds, calls are produced through the syrinx, which is located at the base of the trachea. Syringeal constriction functionally overlaps the role of the larynx in mammalian phonation [20], and the trachea acts as a filter to remove certain frequencies or leave others unchanged. Overall, the source-filter theory can be used to investigate how acoustic variation in animals originates from individual distinctiveness in the morphology and size of the vocal apparatus [21] or voluntary tuning of the vocal organs [22]. For these similarities, the source-filter theory has recently emerged, beyond mammals, as the dominant theory for also explaining the acoustic output of songbirds [23], non-songbirds [24] and even reptiles [25]. Moreover, the source-filter theory predicts that indexical information, such as body size, can be encoded in vocalisations when acoustic features are constrained by the morphology of the vocal structures that are dependent on the growth of the rest of the body [19].

Penguins (Aves, Sphenisciformes, Spheniscidae) are a family of colonial seabirds where vocalisations play a role to maintain group cohesion, mitigate the agonistic encounters [26,27] and, above all, allow recognition between mates and between parents and offspring [28]. Four common vocalisation types can be distinguished in the vocal repertoire of all the species. Namely, a contact call emitted by isolated birds, an agonistic call used in aggressive interactions, an ecstatic display song uttered by single birds during the breeding season, and a mutual display song vocalised by pairs at their nests [26,27,29]. The role of learning in penguin call development is thought to be absent, but the acoustic features of vocalisations are species-specific [30,31] and have evolved under different environmental constraints, ecological pressures, sexual and social sources of selection [26,32]. Moreover, the mechanisms used to encode individual identity information in vocalisations vary widely according to breeding ecology (i.e. nesting vs non-nesting) of the different species [33,34]. Accordingly, it has been recently demonstrated that studying the anatomical constraints that influence nesting penguin vocalisations from a source- filter perspective, can lead to a much better understanding of the acoustic cues of individuality contained in their calls [10,31]. Finally, in a few penguin species, the display songs have been demonstrated to provide acoustic cues to the body size and condition of the emitter. For example, during the breeding period, males with a bigger skeletal size of the little penguin (*Edyptula minor*) give vocalisations at lower dominant frequencies, with females showing more interest for these larger individuals [35]. Similarly, the ecstatic display songs of the Adélie penguins (*Pygoscelis adeliae*) honestly predict the condition and breeding success of the males [36]. Especially for the male penguins, body size and mass are indicators of their desirability as a mate. In territorial species, they can also measure the fighting ability. Accordingly, we hypothesise that the body size and mass of a penguin can affect its vocal organs, which in turn influence the acoustic features of the vocal output.

The genus *Spheniscus* (banded penguins) comprises four extant penguin species that inhabit temperate and equatorial areas of the Southern Hemisphere [37]. Banded penguins mostly breed in large colonies and share similar nesting behaviours [32]. All species build nests in underground burrows that they excavate or use natural depressions [38,39]. Among the different call types, the ecstatic display song, given during the breeding period [29], is the loudest and more complex vocalisation in the repertoire of these species (S1 Video). The song is composed of a sequence of acoustically distinct vocal units (syllables) combined into a phrase [27]. Playbacks experiments of calls demonstrated that Magellanic penguins show individual recognition based on the display songs [40]. In particular, females respond more strongly to ecstatic display songs of mates versus neighbours and strangers. Similarly, the ecstatic display song of the African penguin (*Spheniscus demersus*) has the potential to encode the individual identity information, and both source- and filter-related vocal components are responsible for the individual distinctiveness [10]. Nevertheless, the acoustic features of calls that encode the individual identity information in the Magellanic penguin and even whether the ecstatic display song has the potential to allow individual discrimination in the Humboldt penguin still remain to be investigated.

The main goal of the study is to provide the first comprehensive acoustic analysis of the display songs of the Humboldt and Magellanic penguins. In particular, we address two key questions: (1) Do fundamental frequency and formants have the potential for individual recognition? (2) Does any vocal parameter explain variance in penguins' body size or mass? Overall, we predicted that, in *Spheniscus* penguins, the vocal features of the ecstatic display songs encode individual identity and body size information, and that this vocalisation has the potential to play a role as both social and quality signal during the breeding season.

## Materials and methods

### Ethics statement

The research was carried out with permission from the Acquario di Cattolica and Acquario di Genova and conforms to the Ethical Guidelines for the Conduct of Research on Animals by Zoos and Aquariums [41] and with the Guidelines for the Treatment of Animals in Behavioural Research and Teaching [42]. Acoustic recordings were non-invasive and we made every effort to minimize possible disturbance to the penguins during collection of morphological measurements. All applicable international, national, and/or institutional guidelines for the care and use of animals were followed and no specific permissions were required for these activities according to Italian laws.

### Penguins and recordings

We recorded six adult Humboldt penguins (4 males and 2 females) in 2015 at the Acquario di Cattolica, Italy. The colony was composed of 4 males and 8 females, but ecstatic display songs were uttered only by 6 birds during the breeding season. Penguins were housed in an indoor communal exhibit (75 m^2^ including a saltwater pond of 35 m^2^). The colony was established at the Acquario di Cattolica from 2007 to 2009 joining adult penguins from the Schönbrunn Zoo (Austria) and the North of England Zoological Society, Chester (United Kingdom).

Ecstatic display songs of 12 adult Magellanic penguins (5 males and 7 females) were collected from an *ex-situ* colony at the Acquario di Genova, Italy. All birds were recorded in 2015 during the breeding period. The penguins were originally from Argentina (wild individuals stranded due to an oil spill) imported after the rescue at the SELWO Marina Delfinarium (Benalmadena, Spain) and finally moved to Genoa in 2006. The colony was maintained in a communal indoor exhibit of 123 m^2^ including a saltwater pond of 66 m^2^. The exhibit had three concrete walls and one facing the visitor corridor made up of glass panels, which allows a combined vision of open air and underwater penguin activity.

All vocalisations were collected using the all occurrence animal sampling method [43]. Since vocalisations were labelled according to the emitter, it was not possible to collect blind recordings. Ecstatic display songs were recorded at a distance of between 2 and 10 m from the caller with a RØDE NTG2 Super-Cardioid microphone (frequency response 20 Hz to 20 kHz, sensitivity -36dB +/- 2 dB re 1 V/Pa at 1 kHz, max SPL 131dB) mounted on a RØDE PG2 Pistol Grip. The microphone was connected to a TASCAM DR-680 or TASCAM DR-40 digital recorder (44.1 kHz sampling rate) and we made every effort to orientate the microphone towards the calling bird. Acoustic data were originally saved into an internal SD memory card in WAV format (16-bit amplitude resolution) and then moved to a laptop computer for later acoustic analyses.

### Selection of vocalisations

**Fig 1:**
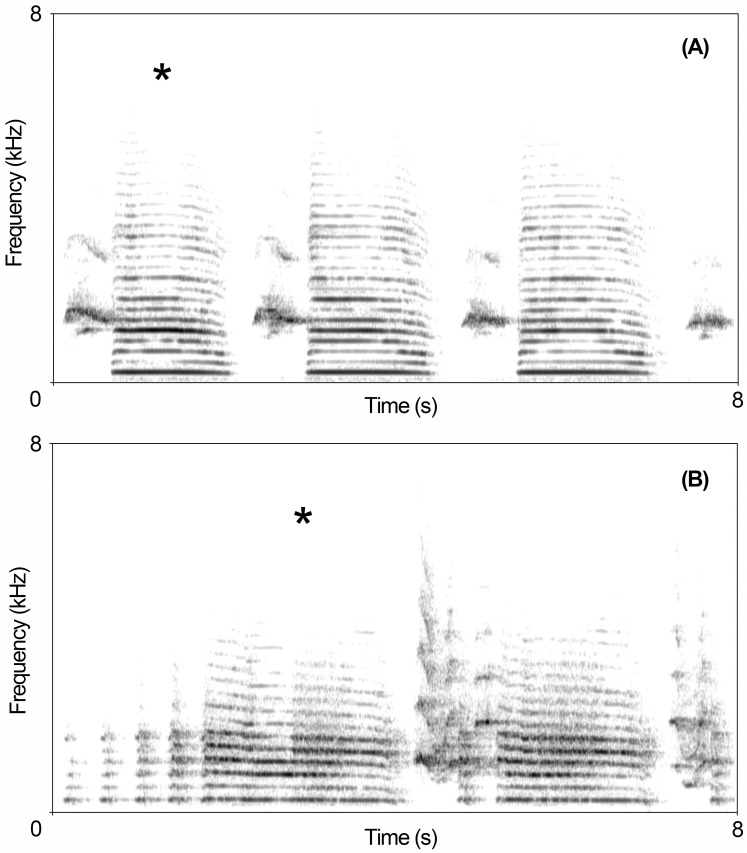
Spectrographic representation of ecstatic display songs uttered by adult Humboldt (A) and Magellanic (B) penguins during the breeding period. Asterisks indicate the syllables type 2. Spectrograms were generated in Praat using a Gaussian window shape, window length = 0.03 s, number of time steps = 1000, number of frequency steps = 250, dynamic range = 50 dB.

The overall spectral structure of audio recordings was visually inspected using a narrow-band spectrogram in Praat v.5.4.0173 [44]. The inspection of vocalisations allowed us to select segments containing the ecstatic display songs (Fig 1) that were saved as separate WAV files. However, approximately 30% of ecstatic display songs for Humboldt and Magellanic penguins were discarded because they showed excessive background noise or because calls were overlapping between different birds vocalising at the same time. Overall, the spectrographic inspection allowed us to select a total of 163 ecstatic display songs for Humboldt penguins and 194 for Magellanic penguins (for the final contribution of each bird see S1 Table). Finally, for ecstatic display song, we identified the longest syllables (i.e. “type 2” according to the terminology used by Favaro et al. [27]; Fig 1). Then, we limited the acoustic analysis to the first long syllable of the song, since vocal features of consecutive calls might be highly correlated.

### Acoustic analysis

Following the source-filter theory approach, we measured eight acoustic parameters for each selected vocalisation using a custom-built script in Praat. The vocal parameters were chosen according to their potential to predict individual and body size distinctiveness. These measurements included temporal measures, such as call duration which is related to lung capacity [45], source-related vocal features (*f*_0_) which are related to the vibrating mass in the syrinx [46], and filter- related features (formants), related to the supra-syringeal vocal tract [18]. Specifically, following the visual inspection of the spectrograms and previous acoustic response of vocal tract models for *Spheniscus* penguins [10,31], we extracted the contour of the first four formants (*F*_1_–*F*_4_) of each vocalisation. We used a Linear Predictive Coding and we set to 5 the maximum number of formants to be tracked by the Praat software below 4000Hz. Lastly, for each call, we calculated the formant dispersion (Δ*F*; [13]) and we estimated the vocal tract length (VTLest) of the caller using the following equation: VTLest = *c / 2ΔF* where c approximates the speed of sound in the vocal tract. The vocal tract was modelled as a uniform tube open at one end (oral cavity) and closed at the other (syrinx). Description and abbreviations for all the acoustic parameters are presented in Table 1.

**Table 1:** Abbreviations and descriptions for the acoustic parameters measured.

Acoustic parameter	Description
Dur (s)	Duration of the vocalisation
*f*_0_ (Hz)	Mean fundamental frequency value across the vocalisation
*F*_1_ (Hz)	Mean frequency values of the first formant across the vocalisation
*F*_2_ (Hz)	Mean frequency values of the second formant across the vocalisation
*F*_3_ (Hz)	Mean frequency values of the third formant across the vocalisation
*F*_4_ (Hz)	Mean frequency values of the fourth formant across the vocalisation
*ΔF* (Hz)	Formant dispersion
VTLest (cm)	Estimated vocal tract length

Vocal parameters were measured only to the first long syllable (i.e. type 2) in the song.

### Morphological measurements

Morphological measurements were taken at the beginning of the breeding period from each penguin, using a digital caliper accurate to 0.005 mm. We collected a total of seven skeletal measurements: homerus width (FL1), ulna length (FL2), carpus + metacarpus + digits length (FL3), bill length (BL, to the culmen), bill width (BW, taken across the center of the culmen), bill depth (BD, taken through the center of the culmen), skull length (SL). We also measured the body mass (weight, W) of the penguins as an indicator of the nutritional status (i.e. fat reserves) of each individual. All the measurements were taken by the same person (LF) in the early morning, before the first feeding session of the day. Penguins were immobilised from the feet and cheek, and measurements were collected from above the animal. All procedures lasted approximately 5 minutes per bird and were supervised by the veterinary staff. Great care was always taken to minimise disturbance to the colonies.

### Statistical analysis

We performed two separate cross-validated (leave-one-out) discriminant function analyses (DFA) for Humboldt and Magellanic penguins to investigate whether the acoustic features of the display songs could allow individual discrimination in these two species. We used the identity of the caller as the group identifier and the acoustic variables as discriminant variables. Normal distribution of discriminant variables was tested (Kolmogorov-Smirnov test) and the data were log-transformed whether necessary. The DFA analyses were performed in SPSS v.22 (IBM Corp. Released 2013. IBM SPSS Statistics for Macintosh, Version 22.0. Armonk, NY: IBM Corp.). In particular, we used a feed forward procedure with the default F-values threshold (i.e. F = 3.84) for acceptance or rejection of the discriminant variables. Finally, the coefficients of classification were corrected according to the group sizes, since the different individuals did not contribute to an equal number of vocalisations.

We built a series of Generalised Linear Mixed Models (GLMMs) using the lme4 package [47] in R v. 3.2.0 [48] to analyse the relationship between vocal parameters and the body size of the penguins. We run each model using one of the vocal parameters as the response variable and log-transformed body measurements as fixed factors. We included species, sex, and emitter identity as random factors. Before running the models, we excluded the occurrence of collinearity among predictors by examining the variance inflation factors (vif package [49]). This procedure allowed us to select five non-collinear predictors (BL, BW, FL1, FL2, W) showing vif < 4. We verified the assumptions that the model residuals were normally distributed and homogeneous by looking at a qqplot and the distribution of the residuals plotted against the fitted values. We then tested the significance of the full model [50] against a null model comprising the random factors exclusively. For this comparison, we used a likelihood ratio test (analysis of variance with argument test “Chisq” [51]). We calculated the P values for the individual predictors based on likelihood ratio tests between the full and the respective null model (R-function “drop1 [52]). We reported estimate, chi-square, and P values for each significant model.

## Results

### Vocal individuality

**Fig 2:**
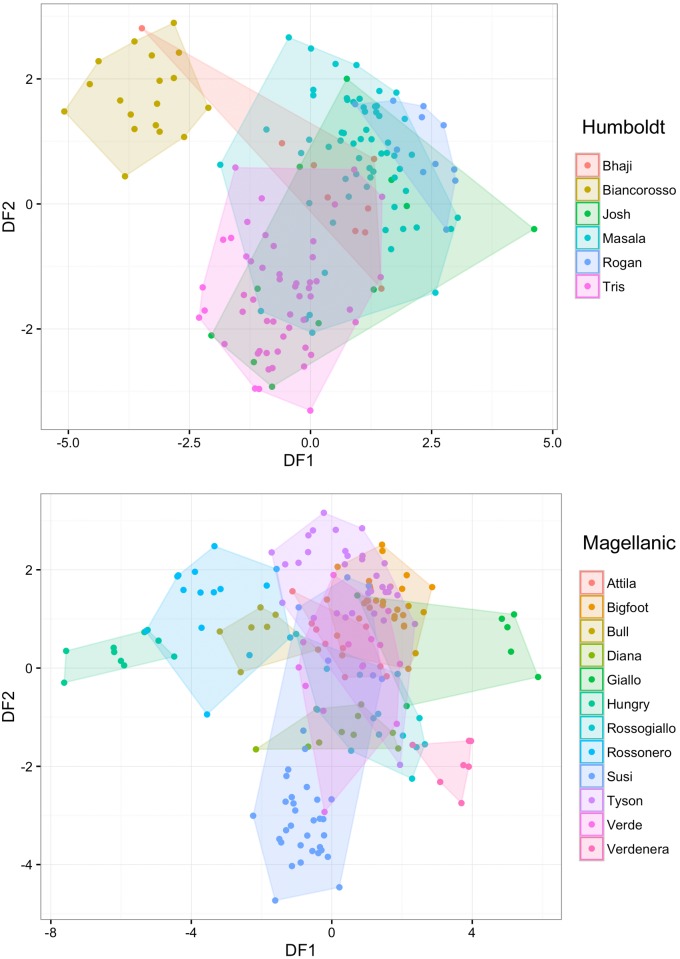
Vocalisations of Humboldt and Magellanic penguins plotted in the two-dimensional space defined by DF1-DF2. The calls of the different individuals made distinctive clusters within the range of variation of their species.

Descriptive statistics for the acoustic parameters measured on vocalisations of each penguin are presented in Table 2. The DFA produced three (see S2 Table for the statistical significance of this classification) discriminant functions (DFs) which could be used to discriminate the different Humboldt penguins with an accuracy of 77.3%. Similarly, for the Magellanic penguins, the DFA produced six statistically significant (S3 Table) discriminant functions that allowed classifying correctly 78.4% of the display songs to the emitter. The accuracy of the DFA decreased to 74.2% for Humboldt and 75.3% for Magellanic penguins, when the more conservative leave-one-out cross-validation was applied. The stepwise procedure was performed in four and six steps for the Humboldt and Magellanic penguins, respectively, and showed that both duration, source- and filter-related acoustic parameters are important for vocal distinctiveness in these two species (Table 3). In the space defined by the DF1 and DF2, the vocalisations of the different individuals made distinctive clusters within the range of variation of their species (Fig 2).

**Table 2:** Name, sex and values of the vocal parameters (mean ± SD) of each Humboldt (a) and Magellanic (b) penguin.

**(a)**		**Bhaji**	**Biancorosso**	**Josh**	**Masala**	**Rogan**	**Tris**						
	Sex	F	F	M	M	M	M						
	N	9	18	11	62	11	52						
	Dur (s)	1.68±0.09	1.59±0.17	1.67±0.54	2.01±0.26	2.38±0.2	1.38±0.2						
	*f*_0_ (Hz)	231±12	210±4	238±7	233±7	240±8	234±6						
	*F*_1_ (Hz)	838±63	884±70	837±181	854±98	838±70	862±121						
	*F*_2_ (Hz)	1532±102	1636±109	1468±89	1495±100	1495±74	1419±110						
	*F*_3_ (Hz)	2268±70	2404±123	2281±80	2240±113	2269±110	2294±98						
	*F*_4_ (Hz)	3053±112	3250±81	3122±112	3081±71	3109±92	3109±89						
	*ΔF* (Hz)	908±30	966±36	917±34	907±30	915±33	913±34						
	VTLest (cm)	19.29±0.64	18.14±0.7	19.12±0.77	19.31±0.63	19.15±0.69	19.19±0.73						
**(b)**		**Attila**	**Bigfoot**	**Bull**	**Diana**	**Giallo**	**Hungry**	**Rossogiallo**	**Rossonero**	**Susi**	**Tyson**	**Verde**	**Verdenera**
	Sex	F	M	F	F	F	M	F	M	F	M	M	F
	N	11	31	7	10	7	14	14	15	37	32	13	7
	Dur (s)	1.55±0.12	1.76±0.19	1.29±0.11	0.98±0.08	1.8±0.23	1.01±0.12	1.22±0.11	1.4±0.19	0.9±0.37	1.93±0.25	1.41±0.29	1.25±0.1
	*f*_0_ (Hz)	261±8	278±11	257±7	300±22	305±23	224±5	284±9	248±11	279±12	259±13	274±7	301±7
	*F*_1_ (Hz)	960±73	906±56	822±57	759±112	973±42	764±24	919±161	693±85	787±117	977±77	877±50	923±17
	*F*_2_ (Hz)	1507±38	1365±50	1378±30	1356±62	1402±71	1381±19	1492±125	1365±34	1445±50	1485±71	1440±61	1504±66
	*F*_3_ (Hz)	2202±85	1991±88	1997±63	2040±148	2087±128	2089±89	2112±105	2050±69	2107±98	2272±146	2159±59	2038±73
	*F*_4_ (Hz)	3051±104	2916±74	2987±58	3019±74	2955±104	3008±69	3030±113	3005±73	2979±81	3104±113	3023±77	2894±94
	*ΔF* (Hz)	901±29	842±25	854±17	861±35	864±35	867±21	885±41	859±20	869±26	917±41	885±22	854±28
	VTLest (cm)	19.44±0.62	20.8±0.6	20.51±0.42	20.36±0.85	20.28±0.79	20.2±0.5	19.82±0.95	20.38±0.47	20.15±0.61	19.12±0.9	19.79±0.48	20.5±0.67

**Table 3:** Standardised coefficients for the canonical discriminant functions generated by the stepwise procedure to classify vocalisations of Humboldt (a) and Magellanic (b) penguins.

	Vocal parameter	Discriminant function					
		1	2	3	4	5	6
(a)	Dur	**0.600**	**0.649**	0.520			
	*f*_0_	**0.785**	-0.478	0.039			
	*F*_2_	0.058	**0.548**	**-1.295**			
	*F*_4_	-0.412	-0.173	**1.506**			
(b)	Dur	0.489	**0.847**	-0.007	0.112	**0.512**	-0.13
	*f*_0_	**1.017**	-0.285	-0.19	0.395	0.098	-0.027
	*F*_1_	0.447	0.022	-0.036	-0.483	**-0.987**	0.355
	*F*_2_	0.35	-0.413	**0.666**	**-0.599**	**1.009**	0.294
	*F*_3_	0.283	-0.274	**0.778**	**0.661**	**-0.585**	**-1.201**
	*F*_4_	**-0.586**	**0.528**	**-0.565**	**0.724**	0.099	**1.211**

Bold text indicates the factor loadings > ±0.5.

### Acoustic cues to body size and mass

**Fig 3:**
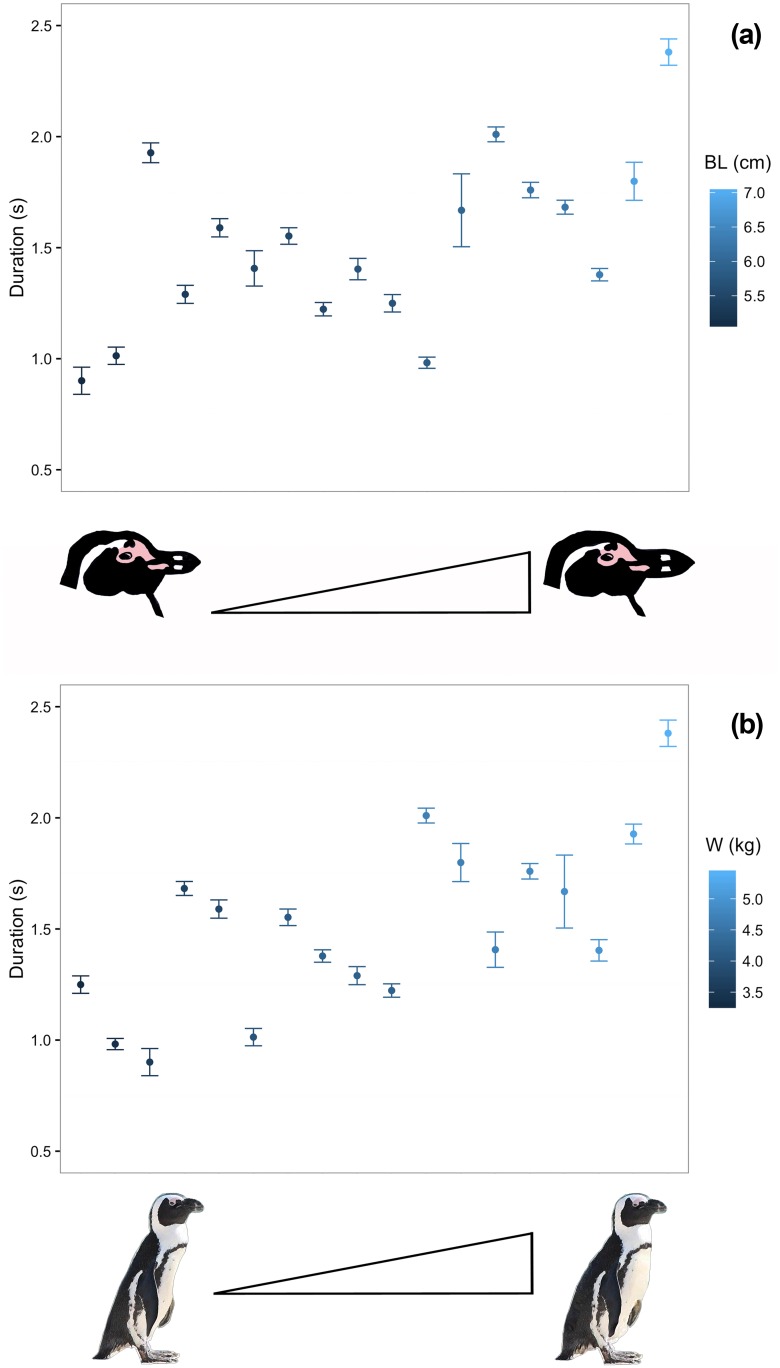
Average values for the bill length (a) and body weight (b) of each penguin plotted against duration of the type 2 syllables. Error bars show 95% confidence Interval.

We found that penguins with bigger body size and mass produce display songs with longer syllables type 2. In particular, the GLMM showed a significant positive effect of the factors bill length (BL; *χ*^*2*^ = 8.133, *P* = 0.004, Estimate = 1.889) and body weight (W; *χ*^*2*^ = 10.296, *P* = 0.001, Estimate = 1.866; Fig 3) on the duration of the syllable, with the full model including the fixed factors fitting the data better than the null model including only the random effects (GLMM full vs. null: *χ*^*2*^ = 21.982, d*f* = 5, *P* < 0.001). We also found a significant negative effect of the body mass (W; *χ*^*2*^ = 6.046, Estimate = - 126.149, *P* = 0.014) on the mean fundamental frequency (*f*_0_) of the syllables (GLMM full vs. null: *χ*^*2*^ = 15.195, d*f* = 5, *P* = 0.009), showing that heavier individuals produce low-pitched vocalisations. Finally, we did not find any effect of the fixed factors on the four formants measured, formant dispersion nor estimated vocal tract length of the penguins. The full models did not significantly differ from the null models for any of these vocal parameters.

## Discussion

We investigated whether acoustic cues of individuality, body size, and mass are encoded in the ecstatic display songs of the Humboldt and Magellanic penguins (genus *Spheniscus*). Using a source-filter theory approach [19], we demonstrated that both fundamental frequency (*f*_0_) and formants are essential vocal features to discriminate among individuals. Moreover, we show that only duration and *f*_0_ are reliable indicators of body size and mass, respectively, and not formant frequencies or formant-related vocal parameters.

While the relationship between skeletal size and resonance frequencies of the vocal tract has been documented in a variety of mammal species [13,14,53], our results support a growing body of literature showing that such relationship is not always present in bird vocalisations [24,54]. Overall, we suggest that the link between anatomical constrains and formant frequencies deserve further investigations from a broader range of avian species.

### Vocal individuality indicators

Individual recognition is essential for social interactions and vocalisations are thought to be a key element for the recognition process [26]. Our results demonstrate that the ecstatic display songs of Humboldt and Magellanic penguins are individually distinctive, with more than 75% of vocalisations assigned to the correct individual. The interpretation of the discriminant functions also confirmed that in these two species, the source- and filter- related components are necessary to separate the calls of the different individuals. Previous playback experiments have shown evidence of vocal recognition in a variety of penguin species [33]. However, in *Spheniscus* penguins, evidence from playback of calls is limited to the Magellanic penguin [40], and it was unclear which acoustic parameters encoded the identity information. Moreover, recently, in the African penguin it has been demonstrated that both the source- and filter- related components can encode individual identity information [10]. Overall, our findings support the hypothesis that in nesting penguins, the acoustic cues to identity are the fundamental frequency and the energy distribution across the spectrum [33,55] and that studying the anatomical constraints that influence the vocal output with a source-filter theory approach can lead to a better understanding of the individual identity information encoded in their vocalisations [10,31].

### Body size and body mass indicators

Our results provide the first evidence for banded penguins that the ecstatic display songs uttered during the breeding period encode acoustic cues to the body size of the emitter. We showed that the duration of the type 2 syllables conveys this information. We also found that heavier birds emit longer and low-pitched vocalisations.

According to the source-filter theory, we predicted that the airstream from the lungs sets the vibrating mass of the penguin syrinx (sound source) into vibration. Firstly, we explain the positive size-duration allometry as a result of lung capacity [45]. In particular, bigger penguins can inhale a bigger volume of air during the inspiration phase preceding the emission of the type 2 syllables of the song [27] (see also S1 Video). Secondly, we suggest that individual-specific factors, such as nutrition, can influence both the duration and the average fundamental frequency of the vocalisations. Indeed, in birds, the rate of vibration of the syringeal membranes (*f*_0_) is passively determined by their size, mass, and tension [56] and the hormonal and nutritional status are thought to play a key role in determining their mechanical and functional proprieties [57]. Overall, our results are also in agreement with recent findings in the Adélie penguin [36], where individuals in better body condition were found to emit lower frequency vocalisations at the beginning of the breeding season.

Overall, larger individuals are selected in a variety of different species because they are favoured to compete with conspecifics for mating or territorial defence [58,59]. Heavier individuals are also potentially more fertile [60] and body weight can reveal nutritional condition and foraging success [61]. Penguins are biparental incubators [62] and the energetic investment of both sexes is not limited to the courtship period [63]. In particular, in nesting and territorial species, mates alternate the egg-brooding and chick-rearing duties, protecting their nest or offspring from neighbours and predators, and attacking other birds that wander close to their nest [64,65]. The body mass can also influence the parents’ foraging behaviour and heavier individuals exhibit a greater capacity to properly feed their chicks [66]. For these reasons, mate choice occurs by characteristics that correlate with body size and mass [63]. However, it is doubtful whether individuals can discriminate the degree of morphological differences of a conspecific visually [63] and a few previous studies have suggested that vocalisations play a fundamental role in this selection process [35,36,63]. Overall, our findings support this hypothesis and show that the ecstatic display songs of banded penguins are honest signals in that they provide reliable cues to the body size and mass of the emitter. Further playback experiments are necessary to determine whether 1) banded penguins can perceive such acoustic differences in vocalisations, and 2) mate choice correlates with duration and/or fundamental frequency of the ecstatic display songs.

Finally, our results do not fit as predicted by the source-filter theory in mammals, where there is a strong basis for an expectation of a linkage between the filter-related components of a vocalisation and body size parameters [13]. In particular, we suggest that the length and volume of *Spheniscus* penguins’ vocal tract (determining formants) are independent of the skeletal size. In several avian orders (particularly in larger and territorial species) the lack of this relationship can be explained by the presence of an elongated trachea, in the light of the “size exaggeration hypothesis” [64]. Although the order Sphenisciformes does not exhibit trachea elongation [67], banded penguins show a trachea made of imbricated cartilaginous rings covered by muscles [68]. This anatomical configuration allows this organ to be voluntarily contracted, to markedly change its length [68] across the different vocal types [10]. This mobility of the supra-syringeal vocal tract is likely to explain the lack of relationship between the skeletal size, formants, and formant dispersion.

### Social recognition versus body size information

Throughout the breeding season, Humboldt and Magellanic penguins have to face completely different contexts of social interaction. In early season, penguins have to find their previous mates or to establish new pairs. In this condition, birds have to interact with many unfamiliar individuals of both sexes. In animal societies, this context has been recently suggested to favour the use of quality signals [69]. Our findings support this hypothesis and indicate that social environment of the early breeding period may have favoured selection for the honest cues to body size and weight in the ecstatic display songs, which can be used by penguins to assess the quality of both potential mates and competitors. However, when territory boundaries are settled and pairs established, interactions mostly occur with neighbours and mates [62]. By contrast, this social setting is expected to favour assessment based on social recognition over quality signalling [69]. Moreover, in the late breeding season, the vocal recognition between mates is mediated by the mutual display songs (i.e. the vocalisations that birds give within the nest overlapping in a duet [26,27]) rather than by the ecstatic display songs. Accordingly, we suggest that acoustic cues of individuality encoded in the ecstatic display songs can play a crucial role in mitigation of the agonistic encounters with neighbours, according to a “dear enemy” effect. This phenomenon consists of an increasing familiarity between neighbours aimed to reduce the energy invested on aggressive interactions and to favour increasing of the individual fitness. The “dear enemy” effect has been previously observed in numerous territorial species of birds [70], mammals [71–73] and even invertebrates [74]. Further research, using playback of calls is required to confirm the presence of this phenomenon also in penguin colonies.

## Supporting information

S1 Table Vocal contribution for each penguin. (PDF)

S2 Table Tests for the canonical discriminant functions established to discriminate among individuals in Humboldt penguins. (PDF)

S3 Table Tests for the canonical discriminant functions established to discriminate among individuals in Magellanic penguins. (PDF)

S1 Video Ecstatic display song given by adult Humboldt penguin at the nest. (MOV)
