# Supplementary material for: Acoustic correlates of body size and individual identity in banded penguins
Source: PLoS One. 2017 Feb 15;12(2):e0170001. doi: 10.1371/journal.pone.0170001 (PMC5310857; doi:10.1371/journal.pone.0170001)
Supplement: S1 Table — (PDF) [file pone.0170001.s001.pdf]

Acoustic correlates of body size and individual identity in banded penguins

Livio Favaro*, Marco Gamba, Claudia Gili, Daniela Pessani

* E-mail: livio.favaro@unito.it

S1 Table. Vocal contribution for each penguin.

Species	Name	Sex	Ecstatic display songs
Humboldt penguin (*Spheniscus humboldti*)	Bhaji	F	9
	Biancorosso	F	18
	Josh	M	11
	Masala	M	62
	Rogan	M	11
	Tris	M	52
Magellanic penguin (*Spheniscus magellanicus*)	Attila	F	11
	Bigfoot	M	31
	Bull	F	7
	Diana	F	10
	Giallo	M	7
	Hungry	F	10
	Rossogiallo	F	14
	Rossonero	M	15
	Susi	F	37
	Tyson	M	32
	Verde	M	13
	Verdenara	F	7
