# Supplementary material for: Acoustic correlates of body size and individual identity in banded penguins
Source: PLoS One. 2017 Feb 15;12(2):e0170001. doi: 10.1371/journal.pone.0170001 (PMC5310857; doi:10.1371/journal.pone.0170001)
Supplement: S2 Table — (PDF) [file pone.0170001.s002.pdf]

Acoustic correlates of body size and individual identity in banded penguins

Livio Favaro*, Marco Gamba, Claudia Gili, Daniela Pessani

* E-mail: livio.favaro@unito.it

S2 Table. Tests for the canonical discriminant functions established to discriminate among individuals in Humboldt penguins.

Function	Eigenvalue	Variance explained	Test of Function	Wilks's λ	χ^2	d*f*	Significance
1	2.245	57.9%	1 – 4	0.102	357.829	20	*P* < 0.001
2	1.363	35.2%	2 – 4	0.332	173.040	12	*P* < 0.001
3	0.248	6.4%	3 – 4	0.785	38.036	6	*P* < 0.001
4	0.021	6%	4	0.979	3.313	2	*P* = 0.191
