# Supplementary material for: Acoustic correlates of body size and individual identity in banded penguins
Source: PLoS One. 2017 Feb 15;12(2):e0170001. doi: 10.1371/journal.pone.0170001 (PMC5310857; doi:10.1371/journal.pone.0170001)
Supplement: S3 Table — (PDF) [file pone.0170001.s003.pdf]

Acoustic correlates of body size and individual identity in banded penguins

Livio Favaro*, Marco Gamba, Claudia Gili, Daniela Pessani

* E-mail: livio.favaro@unito.it

S3 Table. Tests for the canonical discriminant functions established to discriminate among individuals in Magellanic penguins.

Function	Eigenvalue	Variance explained	Test of Function	Wilks's λ	χ^2	d*f*	Significance
1	4.776	52.2%	1 – 6	0.12	809.257		*P* < 0.001
2	2.623	28.7%	2 – 6	0.71	486.569		*P* < 0.001
3	0.939	10.3%	3 – 6	0.257	249.687		*P* < 0.001
4	0.488	5.3%	4 – 6	0.499	127.894		*P* < 0.001
5	0.205	2.2%	5 – 6	0.742	54.818		*P* < 0.001
6	0.118	1.3%	6	0.895	20.473		*P* < 0.001
